# Development and Validation of a Simple-to-Use Nomogram to Predict Early Death in Metastatic Pancreatic Adenocarcinoma

**DOI:** 10.3389/fonc.2021.729175

**Published:** 2021-09-09

**Authors:** Zhong Zhang, Juan Pu, Haijun Zhang

**Affiliations:** ^1^Department of Oncology, The Affiliated Zhongda Hospital of Southeast University, Medical School of Southeast University, Nanjing, China; ^2^Department of Oncology, Lianshui People’s Hospital, Huaian, China

**Keywords:** pancreatic adenocarcinoma, SEER database, early death, nomogram, prognosis

## Abstract

**Background:**

Pancreatic adenocarcinoma (PCa) is a highly aggressive malignancy with high risk of early death (survival time ≤3 months). The present study aimed to identify associated risk factors and develop a simple-to-use nomogram to predict early death in metastatic PCa patients.

**Methods:**

Patients diagnosed with metastatic PCa between 2010 and 2015 from the Surveillance, Epidemiology, and End Results (SEER) database were collected for model construction and internal validation. An independent data set was obtained from China for external validation. Independent risk variables contributed to early death were identified by logistic regression models, which were then used to construct a nomogram. Internal and external validation was performed to evaluate the nomogram using calibration curves and the receiver operating characteristic curves.

**Results:**

A total of 19,464 patients in the SEER cohort and 67 patients in the Chinese cohort were included. Patients from the SEER database were randomly divided into the training cohort (n = 13,040) and internal validation cohort (n = 6,424). Patients in the Chinese cohort were selected for the external validation cohort. Overall, 10,484 patients experienced early death in the SEER cohort and 35 in the Chinese cohort. A reliable nomogram was constructed on the basis of 11 significant risk factors. Internal validation and external validation of the nomogram showed high accuracy in predicting early death. Decision curve analysis demonstrated that this predictive nomogram had excellent and potential clinical applicability.

**Conclusion:**

The nomogram provided a simple-to-use tool to distinguish early death in patients with metastatic PCa, assisting clinicians in implementing individualized treatment regimens.

## Introduction

All over the world, pancreatic adenocarcinoma (PCa) remains one of the most lethal cancers and was reported to be the fourth leading cause of cancer-related deaths in 2020 (1). On account of its insidious symptoms and high metastatic potential, more than 50% of patients with PCa are diagnosed at an advanced stage, which results in a dismal 5-year relative survival of 6% (2, 3).

Although most patients with metastatic pancreatic adenocarcinoma (mPCa) have no therapeutic options besides systemic chemotherapy for finite improvement of overall survival (OS), the objective response rates of first-line chemotherapy are less than 50% (4–6). Immunotherapy, which yields brilliant results in other cancers (7, 8), achieves unsatisfactory results in PCa, mostly because of its immunosuppressive tumor microenvironment (9). Thus, patients with mPCa have a poor prognosis and only 19.2% survive beyond 1 year of diagnosis (10), and approximately 50% of patients survive past 3 months according to data collected in the SEER database, a condition defined as early death. Thus, patients with mPCa are prone to early death because of the delayed early diagnosis and restricted outcomes of treatment. Exploration of factors contributing to early death is beneficial for the development of individualized strategies, which can help to improve patient survival and reduce disease burden. To date, there have been no in-depth studies investigating mortality within 3 months of diagnosis of mPCa. Thus, a simple-to-use and predictive model able to distinguish the potential risk factors contributing to early death would be valuable for patients with mPCa.

Tumor incidence and survival data for approximately 34.6% of the US cancer registry population have been recorded in the Surveillance, Epidemiology, and End Results (SEER) database (11). Studies based on a very large multicenter database provide more convincing evidence than single-center studies. Herein, after extracting baseline information, a set of patients with mPCa from the SEER database and an independent Chinese cohort were chosen to recognize factors related to early death and a simple-to-use nomogram was developed for predicting its incidence.

## Methods

### Ethics Approval and Consent to Participate

The authors obtained authorization to exact and analyze the research data stored in the SEER program from the National Cancer Institute, USA (reference number 10528-Nov2020). Informed patient consent was not required to access data through the SEER database. This study was conducted in strict accordance with the 1964 Helsinki Declaration and subsequent amendments or similar ethical standards. This retrospective study of the Chinese cohort was approved by the ethics committee of Zhongda Hospital, Medical School of Southeast University.

### Patient Cohorts and Characteristics

**Figure 1:**
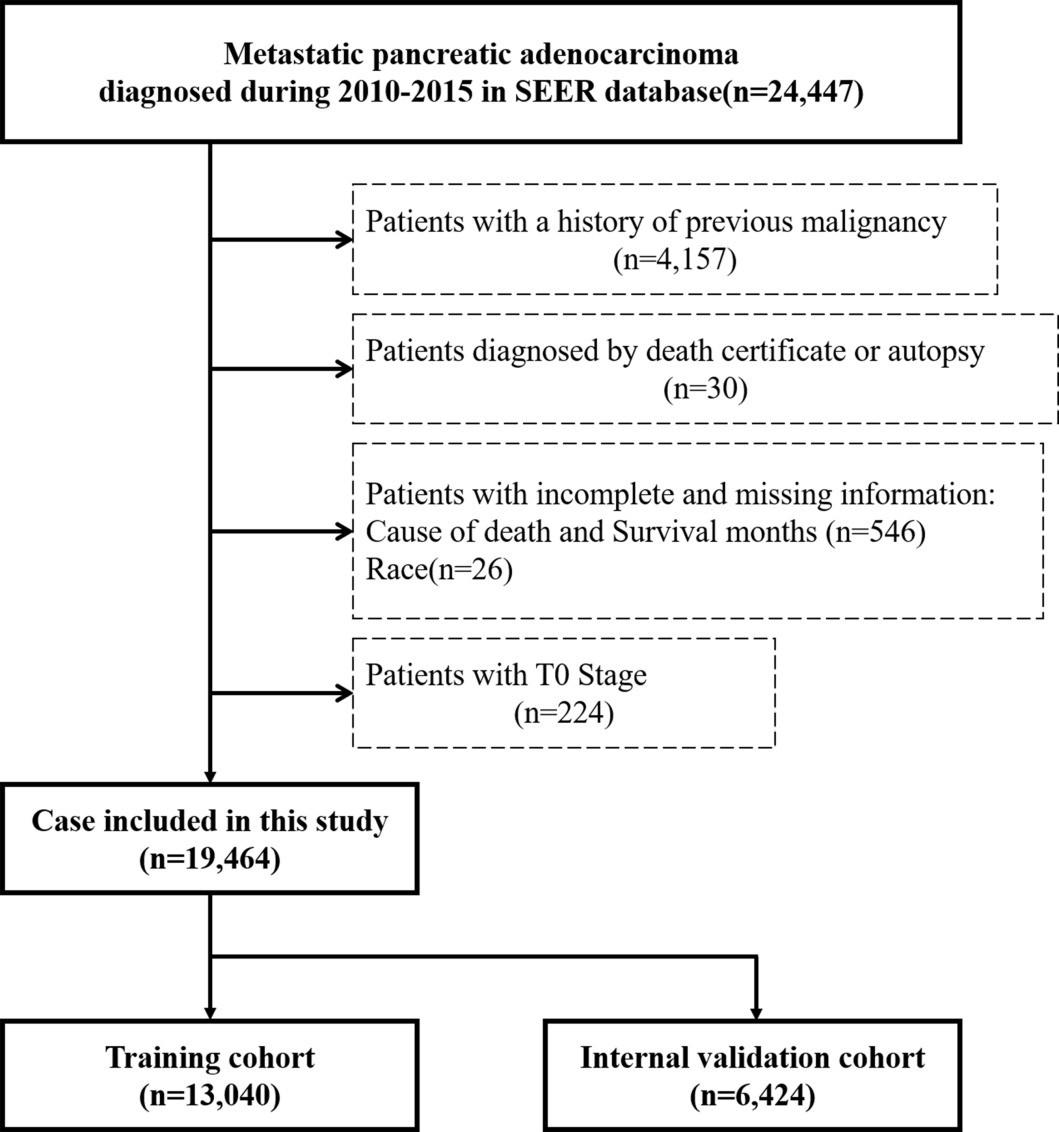
Flow diagram of patient selection criteria. According to the criteria, 19,464 patients were collected from the SEER database and randomly assigned into the training cohort (n = 13,040) and internal validation cohort (n = 6,424).

Data including clinical information were extracted using SEER*Stat version 8.3.9. For this study, we selected patients with stage IV PCa in the SEER database registered from 2010 to 2015. The inclusion criteria were as follows (1): the inclusion site codes C25.0–C25.3 and C25.7–C25.9 and (2) histological codes 8140/3, 8225/3, 8480/3, 8481/3, 8500/3, and 8521/3 [according to the International Classification of Tumor Diseases Third Edition (ICD-O-3)]. The cases who were (1) with a history of previous malignancy (2), diagnosed by death certificate or autopsy, and (3) with T0 stage or incomplete information including cause of death, survival months, and race were excluded. Since surgery was not included in the standard treatment guidelines for mPCa, patients lacking specific information on T stage, N stage, or grade were retained in the cohort. **Figure 1** shows the flow diagram of the patient selection criteria. Considering the malignancy of pancreatic cancer and definition of previous studies, early death was defined as death within 3 months after first diagnosis (12, 13).

Patients from the SEER database were randomized at a ratio of 2:1 and assigned to the training cohort and internal validation cohort, respectively, for the construction and verification of the nomogram. The following demographic and clinical characteristics were collected: age at diagnosis, race, sex, primary site, grade, T stage (American Joint Commission on Cancer [AJCC] 7th version), N stage (AJCC 7th version), liver metastasis, bone metastasis, brain metastasis, lung metastasis, surgery, radiotherapy, chemotherapy, cause of death, vital status, and survival months. For external validation, the same characteristics from another set of patients were collected from Zhongda Hospital, Medical School of Southeast University, between 2014 and 2019, which was selected by the same inclusion and exclusion criteria as SEER cohort.

### Nomogram Development and Statistical Analysis

The basic characteristics of the included patients were described by number and percentage (n, %). Each variable’s contribution in predicting early death of mPCa in the training cohort was tested by univariate logistic analysis. Variables that were statistically significant were further analyzed by multivariate logistic regression. The odds ratios (OR) with corresponding 95% confidence interval (CI) was calculated. Risk factors which were statistically significant in the multivariate analysis were used to construct a predictive nomogram to predict the risk of early death. Nomogram performance was evaluated with respect to discrimination and calibration. For discrimination ability, the nomogram was evaluated using the area under the receiver operating characteristic (ROC) curve (AUC) (14). Calibration curves were plotted to verify the accuracy and reliability of the nomogram (15). Internal and external validations were performed to validate the nomogram. Moreover, decision curve analysis (DCA) was plotted to measure the applicability of the nomogram to clinical practice (16, 17). All statistical analyses were performed with SPSS v 20.0 (IBM Corp., Armonk, NY, USA) and R software v3.6.4 (https://www.r-project.org/). Statistically significance was considered for two-sided p-values <0.01. The R packages rms, pROC, and rmda were applied for data processing.

## Results

### Demographic and Clinical Characteristics

A total of 19,464 patients with mPCa were enrolled from the SEER database in this study, among which 10,484 patients experienced early death. A total of 10,133 patients experienced early death because of PCa. Thus, 13,040 patients were divided into the training cohort and 6,424 were divided into the internal validation cohort. The bulk of early death appeared in participants who were of male sex (54.8%), white (78.3%), and aged between 65 and 79 years (45.0%). The pancreatic head (31.2%) was the most common location associated with early death among patients. Except for the unknown grades (80.8%), the rate of early death in poorly/undifferentiated mPCa versus well/moderately mPCa was 11.5% versus 7.7%. The most common site for metastasis in patients with early death was the liver (78.4%). With regard to treatment, most patients with early death were not treated surgically (96.5%), and only a few patients received radiotherapy (3.5%) or chemotherapy (31.9%). As for the external validation cohort, 67 patients from our center were included in this study, with 35 patients experiencing early death. Similarly, most patients (51.4%) with early death were between 45 and 79 years, and 51.4% of them were male. Liver metastasis (94.3%) was the most common metastatic site. **Table 1** shows the incidence of early death in patients with mPCa, and **Table 2** summarizes the demographic and clinical characteristics of the mPCa patients in the training cohort, internal validation cohort, and external validation cohort.

**Table 1:** Early death events in patients with mPCa.

Characteristic	SEER cohort (n = 19,464)		Chinese cohort (n = 67)	
	Early death (%)	No early death (%)	Early death (%)	No early death (%)
**All**	10,484 (53.9)	8,980 (46.1)	35 (52.2)	32 (47.8)
**Age (years)**				
<50	415 (4.0)	692 (7.7)	3 (8.6)	1 (3.1)
50–64	3,336 (31.8)	3,676 (40.9)	8 (22.9)	13 (40.6)
65–79	4,723 (45.0)	3,785 (42.1)	18 (51.4)	16 (50.0)
≥80	2,010 (19.2)	827 (9.2)	6 (17.1)	2 (6.3)
**Sex**				
Female	4,743 (45.2)	4,260 (47.4)	17 (48.6)	11 (34.4)
Male	5,741 (54.8)	4,720 (52.6)	18 (51.4)	21 (65.6)
**Race**				
White	8,213 (78.3)	7,162 (79.8)	0 (0.0)	0 (0.0)
Black	1,454 (13.9)	1,110 (12.4)	0 (0.0)	0 (0.0)
Other^a^	817 (7.8)	708 (7.9)	35 (100.0)	32 (100.0)
**Primary site**				
Head	3,270 (31.2)	3,522 (39.2)	11 (31.4)	11 (34.4)
Body	1,559 (14.9)	1,608 (17.9)	8 (22.9)	8 (25.0)
Tail	2,338 (22.3)	1,698 (18.9)	11 (31.4)	13 (40.6)
Other^b^	3,317 (31.6)	2,152 (24.0)	5 (14.3)	0 (0.0)
**Grade**				
Well/moderately	808 (7.7)	1,105 (12.3)	2 (5.7)	7 (21.9)
Poorly/undifferentiated	1,206 (11.5)	928 (10.3)	6 (17.1)	5 (15.6)
Unknown	8,470 (80.8)	6,947 (77.4)	27 (77.1)	20 (62.5)
**T stage**				
T1	237 (2.3)	240 (2.7)	2 (5.7)	0 (0.0)
T2	2,631 (25.1)	2,176 (24.2)	6 (17.1)	5 (15.6)
T3	2,563 (24.4)	2,738 (30.5)	5 (14.3)	12 (37.5)
T4	1,748 (16.7)	1,946 (21.7)	13 (37.1)	12 (37.5)
TX	3,305 (31.5)	1,880 (20.9)	9 (25.7)	3 (9.4)
**N stage**				
N0	5,082 (48.5)	4,508 (50.2)	5 (14.3)	5 (15.6)
N1	3,140 (30.0)	3,147 (35.0)	19 (54.3)	12 (37.5)
NX	2,262 (21.6)	1,325 (14.8)	11 (31.4)	15 (46.9)
**Liver metastasis**				
No	2,027 (19.3)	2,537 (28.3)	2 (5.7)	0 (0.0)
Yes	8,215 (78.4)	6,269 (69.8)	33 (94.3)	32 (100)
Unknown	242 (2.3)	174 (1.9)	0 (0.0)	0 (0.0)
**Bone metastasis**				
No	9,113 (86.9)	8,126 (90.5)	8 (22.9)	17 (53.1)
Yes	847 (8.1)	521 (5.8)	7 (20.0)	1 (3.1)
Unknown	524 (5.0)	333 (3.7)	20 (57.1)	14 (43.8)
**Brain metastasis**				
No	9,816 (93.6)	8,598 (95.7)	30 (85.7)	31 (96.9)
Yes	95 (0.9)	29 (0.3)	0 (0.0)	0 (0.0)
Unknown	573 (5.5)	353 (3.9)	5 (14.3)	1 (3.1)
**Lung metastasis**				
No	7,523 (71.8)	6,888 (76.7)	25 (71.4)	29 (90.6)
Yes	2,363 (22.5)	1,706 (19.0)	9 (25.7)	2 (6.3)
Unknown	598 (5.7)	386 (4.3)	1 (2.9)	1 (3.1)
**Surgery**				
No	10,356 (98.8)	8,589 (95.6)	34 (97.1)	31 (96.9)
Yes	90 (0.9)	345 (3.8)	1 (2.9)	1 (3.1)
Unknown	38 (0.4)	46 (0.5)	0 (0.0)	0 (0.0)
**Radiotherapy**				
No/unknown	10,132 (96.6)	8,325 (92.7)	32 (91.4)	14 (43.8)
Yes	352 (3.4)	655 (7.3)	3 (8.6)	18 (56.3)
**Chemotherapy**				
No/unknown	7,141 (68.1)	1,643 (18.3)	26 (74.3)	12 (37.5)
Yes	3,343 (31.9)	7,337 (81.7)	9 (25.7)	20 (62.5)
**Cause of death**				
PCa	10,133 (96.7)	8,730 (97.2)	–	–
Other causes	351 (3.3)	250 (2.8)	–	–

a American Indian/AK Native, Asian/Pacific Islander, Asian.

b Code C25.3 and C25.7–C25.9.

**Table 2:** Demographic and clinical characteristics of the training cohort, internal validation cohort, and external validation cohort.

Characteristics	Training cohort (n = 10,340)	Internal validation cohort (n = 6,424)	External validation cohort (n = 67)
	No. (%)	No. (%)	No. (%)
**Age (years)**			
<50	738 (5.7)	369 (5.7)	4 (6.0)
50–64	4,708 (36.1)	2,304 (35.9)	21 (31.3)
65–79	5,730 (43.9)	2,778 (43.2)	34 (50.7)
≥80	1,864 (14.3)	973 (15.1)	8 (11.9)
**Sex**			
Female	6,044 (46.3)	2,959 (46.1)	28 (41.8)
Male	6,996 (53.7)	3,465 (53.9)	39 (58.2)
**Race**			
White	10,355 (79.4)	5,020 (78.1)	0 (0.0)
Black	1,704 (13.1)	860 (13.4)	0 (0.0)
Other^a^	981 (7.5)	544 (8.5)	0 (0.0)
**Primary site**			
Head	4,543 (34.8)	2,249 (35.0)	22 (32.8)
Body	2,162 (16.6)	1,005 (15.6)	16 (23.9)
Tail	2,673 (20.5)	1,363 (21.2)	24 (35.8)
Other^b^	3,662 (28.1)	1,807 (28.1)	5 (7.5)
**Grade**			
Well/moderately	1,240 (9.5)	673 (10.5)	9 (13.4)
Poorly/undifferentiated	1,441 (11.1)	693 (10.8)	11 (16.4)
Unknown	10,359 (79.4)	5,058 (78.7)	47 (70.1)
**T stage**			
T1	319 (2.4)	158 (2.5)	2 (3.0)
T2	3,236 (24.8)	1,571 (24.5)	13 (19.4)
T3	3,557 (27.3)	1,744 (27.1)	23 (34.3)
T4	2,482 (19.0)	1,212 (18.9)	29 (43.3)
TX	3,446 (26.4)	1,739 (27.1)	0 (0.0)
**N stage**			
N0	6,416 (49.2)	3,174 (49.4)	13 (19.4)
N1	4,242 (32.5)	2,045 (31.8)	54 (80.6)
NX	2,382 (18.3)	1,205 (18.8)	0 (0.0)
**Liver metastasis**			
No	3,075 (23.6)	1,489 (23.2)	2 (3.0)
Yes	9,691 (74.3)	4,793 (74.6)	65 (97.0)
Unknown	274 (2.1)	142 (2.2)	0 (0.0)
**Bone metastasis**			
No	11,544 (88.5)	5,695 (88.6)	25 (37.3)
Yes	936 (7.2)	432 (6.7)	8 (11.9)
Unknown	560 (4.3)	297 (4.6)	34 (50.7)
**Brain metastasis**			
No	12,338 (94.6)	6,076 (94.6)	61 (91.0)
Yes	88 (0.7)	36 (0.6)	0 (0.0)
Unknown	614 (4.7)	312 (4.9)	6 (9.0)
**Lung metastasis**			
No	9,623 (73.8)	4,788 (74.5)	54 (80.6)
Yes	2,761 (21.2)	1,308 (20.4)	11 (16.4)
Unknown	656 (5.0)	328 (5.1)	2 (3.0)
**Surgery**			
No	12,704 (97.4)	6,241 (97.1)	65 (97.0)
Yes	275 (2.1)	160 (2.5)	2 (3.0)
Unknown	61 (0.5)	23 (0.4)	0 (0.0)
**Radiotherapy**			
No/unknown	12,349 (94.7)	6,108 (95.1)	46 (68.7)
Yes	691 (5.3)	316 (4.9)	21 (31.3)
**Chemotherapy**			
No/unknown	5,816 (44.6)	2,968 (46.2)	38 (56.7)
Yes	7,224 (55.4)	3,456 (53.8)	29 (43.3)
**Early death**			
No	5,994 (46.0)	2,986 (46.5)	32 (47.8)
Yes	7046 (54.0)	3,438 (53.5)	35 (52.2)

a American Indian/AK Native, Asian/Pacific Islander, Asian.

b Code C25.3 and C25.7–C25.9.

### Risk Factors for Early Death in the Training Cohort

To further analyze candidate risk factors for early death of mPCa, a binary logistic regression analysis for variables associated with early death is shown in **Table 3**. Univariate logistic analysis revealed that age, sex, primary tumor site, grade, T stage, N stage, liver metastasis, bone metastasis, brain metastasis, lung metastasis, surgery, radiotherapy, and chemotherapy were significantly related to early death. In addition, analysis using a multivariate logistic model identified 11 independent risk factors associated with the early death of mPCa, which included age, sex, primary tumor site, grade, liver metastasis, bone metastasis, brain metastasis, lung metastasis, surgery, radiotherapy, and chemotherapy.

**Table 3:** Univariate and multivariate logistic regression for analyzing the risk factors for early death of mPCa in the training cohort.

Characteristic	Univariate			Multivariate		
	OR	95% CI	*p* value	OR	95% CI	*p* value
**Age (years)**						
<50	Ref			Ref		
50–64	1.517	1.293–1.779	**<0.001**	1.292	1.074–1.554	**0.007**
65–79	2.101	1.794–2.460	**<0.001**	1.585	1.320–1.903	**<0.001**
≥80	4.113	3.437–4.922	**<0.001**	2.020	1.636–2.494	**<0.001**
**Sex**						
Female	Ref			Ref		
Male	1.103	1.029–1.182	**0.005**	1.217	1.120–1.323	**<0.001**
**Race**						
White	Ref			–		
Black	1.096	0.988–1.215	0.083	–	–	–
Other^a^	1.040	0.912–1.186	0.559	–	–	–
**Primary site**						
Head	Ref			Ref		
Body	1.028	0.928–1.139	0.592	1.084	0.959–1.225	0.197
Tail	1.412	1.282–1.554	**<0.001**	1.430	1.275–1.604	**<0.001**
Other^b^	1.695	1.552–1.852	**<0.001**	1.449	1.300–1.614	**<0.001**
**Grade**						
Well/moderately	Ref			Ref		
Poorly/undifferentiated	1.861	1.596–2.171	**<0.001**	1.787	1.487–2.147	**<0.001**
Unknown	1.708	1.516–1.924	**<0.001**	1.424	1.232–1.645	**<0.001**
**T stage**						
T1	Ref			Ref		
T2	1.163	0.924–1.464	0.199	1.273	0.970–1.672	0.082
T3	0.890	0.708–1.119	0.320	1.092	0.833–1.432	0.525
T4	0.943	0.747–1.191	0.621	1.170	0.888–1.543	0.265
TX	1.694	1.346–2.133	**<0.001**	1.418	1.078–1.867	0.013
**N stage**						
N0	Ref			Ref		
N1	0.893	0.827–0.965	**0.004**	1.037	0.944–1.140	0.448
NX	1.470	1.335–1.618	**<0.001**	1.098	0.972–1.240	0.132
**Liver metastasis**						
No	Ref			Ref		
Yes	1.694	1.561–1.839	**<0.001**	1.850	1.673–2.046	**<0.001**
Unknown	1.656	1.290–2.124	**<0.001**	1.540	1.091–2.175	0.014
**Bone metastasis**						
No	Ref			Ref		
Yes	1.482	1.292–1.701	**<0.001**	1.674	1.412–1.984	**<0.001**
Unknown	1.338	1.125–1.591	**<0.001**	0.972	0.658–1.437	0.888
**Brain metastasis**						
No	Ref			Ref		
Yes	2.951	1.790–4.865	**<0.001**	3.037	1.668–5.531	**<0.001**
Unknown	1.343	1.138–1.586	**<0.001**	0.844	0.584–1.219	0.366
**Lung metastasis**						
No	Ref			Ref		
Yes	1.236	1.134–1.346	**<0.001**	1.242	1.119–1.379	**<0.001**
Unknown	1.367	1.163–1.606	**<0.001**	0.977	0.750–1.272	0.860
**Surgery**						
No	Ref			Ref		
Yes	0.206	0.153–0.277	**<0.001**	0.259	0.182–0.366	**<0.001**
Unknown	0.655	0.394–1.086	0.101	0.528	0.280–0.995	0.048
**Radiotherapy**						
No/unknown	Ref			Ref		
Yes	0.439	0.374–0.516	**<0.001**	0.543	0.444–0.664	**<0.001**
**Chemotherapy**						
No/unknown	Ref			Ref		
Yes	0.101	0.093–0.110	**<0.001**	0.106	0.097–0.116	**<0.001**

a American Indian/AK Native, Asian/Pacific Islander.

b Code C25.3 and C25.7–C25.9.

The bold values mean statistically significance (p < 0.01).

### Nomogram Construction

**Figure 2:**
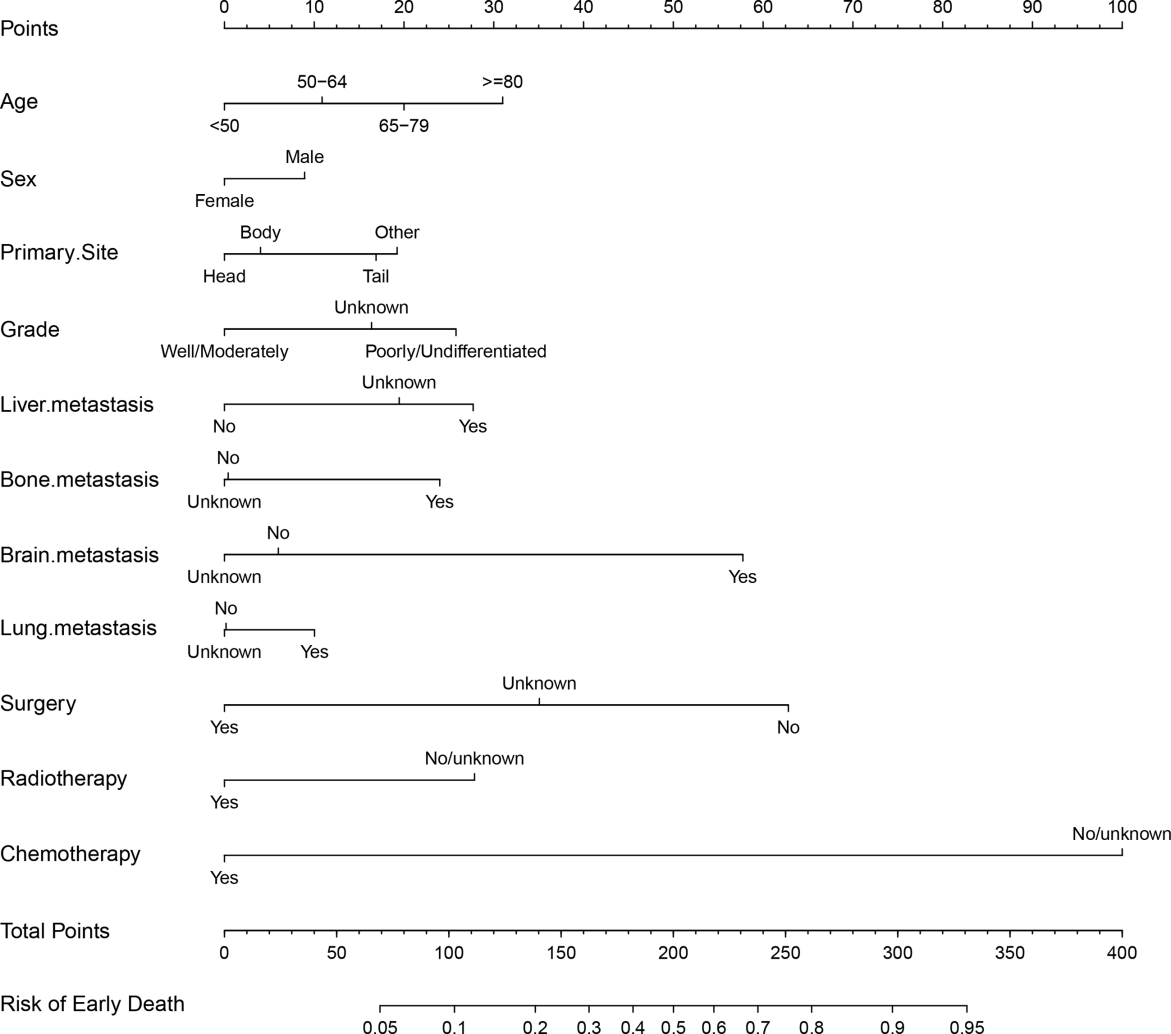
The nomogram of early death in patients with metastatic pancreatic adenocarcinoma.

Based on the significant and independent risk factors identified by the multivariate analysis, a nomogram for predicting early death of mPCa was constructed (**Figure 2**). Chemotherapy contributed to early death mostly and was followed by surgery, brain metastasis, age, and radiotherapy according to the nomogram. In the tool, the total point score was obtained by summing the calculated points of each variable; thus, the probability of early death in mPCa was estimated simply. For example, a male diagnosed at age 70 years with pancreatic tail and liver metastasis, with poorly differentiated grade and who had received only chemotherapy, had a predicted probability of early death of approximately 48% using this nomogram.

### Performance and Validation of the Nomogram

**Figure 3:**
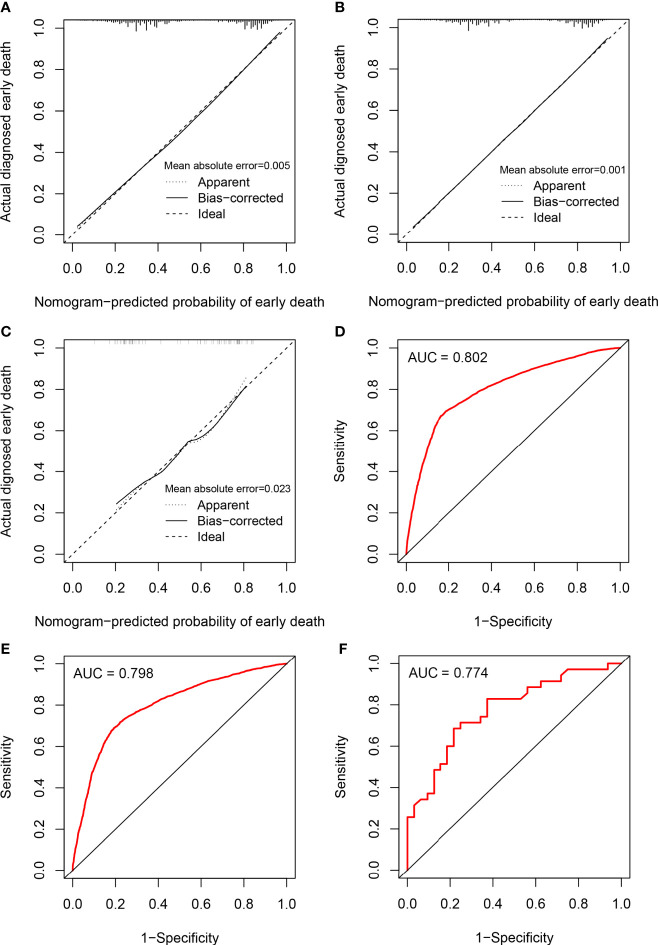
Calibration plots and ROC curves for the nomogram. Calibration plots for the nomogram in **(A)** the training cohort, **(B)** the internal validation cohort, and **(C)** the external validation cohort; ROC curves for the nomogram in **(D)** the training cohort, **(E)** the internal validation cohort, and **(F)** the external validation cohort.

The calibration of the model was assessed using calibration curves. **Figure 3A** shows the high uniformity between the predicted and actual probability of early death in the training cohort, which was then validated using the internal and external validation cohorts (**Figures 3B, C**). The prediction curve was always accompanied by a curve indicating the actual probability. In addition, ROC analysis was used to evaluate predictive efficiencies for early death of the nomogram model. The ROC curves of early death in the training cohort (**Figure 3D**), the internal validation cohort (**Figure 3E**), and the external validation cohort (**Figure 3F**) presented excellent discrimination. Meanwhile, the AUC values of the nomogram were 0.802 (95% CI 0.795–0.810), 0.798 (95% CI 0.787–0.808), and 0.774 (95% CI 0.662–0.886), respectively. As a result of the discrimination test, this nomogram showed reliable predictive ability for early death events.

### Clinical Utility

**Figure 4:**
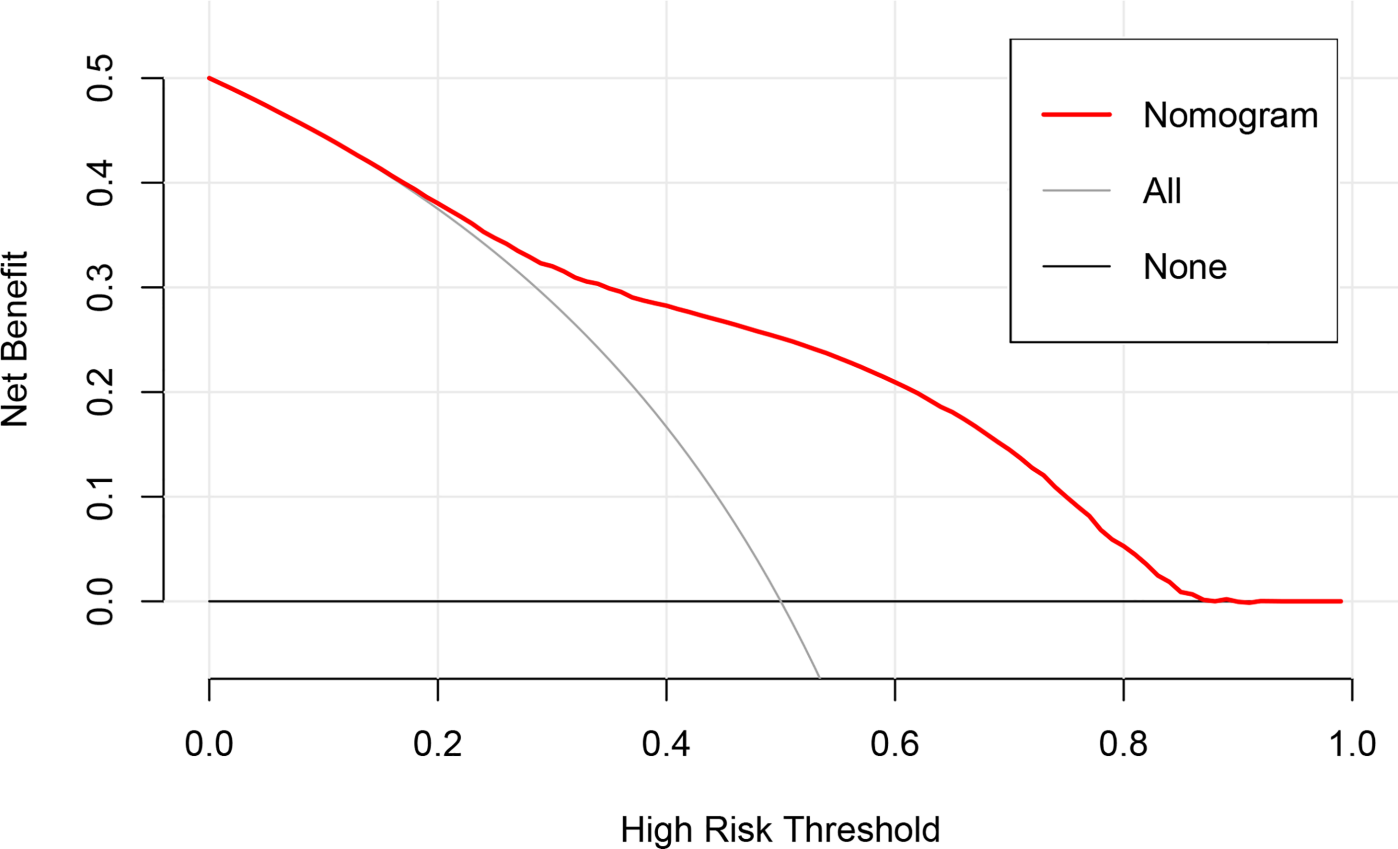
The decision curve analysis curve for the nomogram.

To evaluate the clinical applicability of the nomogram, DCA (**Figure 4**), an advanced method for analyzing the net clinical benefits of predictive models showed that the most favorable threshold probability for predicting early death in the training cohort with the nomogram was 0.2–0.8. As demonstrated by the favorable threshold probability, it indicated that the nomogram could assist clinicians to assess early death of mPCa patients accurately.

## Discussion

PCa is a significant public health problem and is characterized by a high mortality rate with increasing incidence worldwide (18). Improvements in median survival based on standard treatment regimens have not been satisfactory compared to other cancers (4, 19–21). According to the SEER database, more than 50% of patients with mPCa experience early death, defined as survival ≤ 3 months. Most studies on the prognosis of PCa have focused on the long-term survival of patients (22, 23) and risk factors associated with early mortality in resectable pancreatic adenocarcinoma (24, 25). However, advanced tumors have a higher risk of early death, and the assessment of risk factors for early death in mPCa to establish individualized treatment regimens is necessary. Furthermore, a systematic review of phase III trial studies for advanced pancreatic cancer mentioned that early mortality in patients with advanced pancreatic cancer was 23.3%, possibly due to essentially underreported vascular thromboembolic events (26). If it were possible to predict patients at high risk of early death, this could be useful to provide information on the utility of targeted thromboprophylaxis and possibly improve the poor prognosis. For example, the best supportive therapy could be provided depending on the patient’s performance status. Moreover, distinguishing patients with high risk of early death is beneficial for developing clinical trials for advanced pancreatic cancer. Therefore, in this study, we established a predictive nomogram on the basis of independent risk factors to recognize whether patients with mPCa experienced early death.

Demographic information, including age, sex, and race, were identified to be intimately related to the prognosis of mPCa (27–29). In this study, the nomogram also evaluated the impact of these demographic characteristics on early death of mPCa. Among these, and consistent with previous studies, age and sex were significantly associated with early death of mPCa, but the contribution of race was not observed. Apart from demographic factors, early death of mPCa was mainly related to clinical factors such as histological staging, distant metastasis, and therapies including surgery, radiotherapy, and chemotherapy. Surgery has an important impact on the improvement of early death in mPCa, whereas some studies have emphasized the beneficial value of surgery in advanced, and especially, in oligometastatic pancreatic cancer (30, 31). However, considering the small number of patients that had received surgery in our study, it might be more prudent to set strict indications for surgery in mPCa based on the clinical conditions of the patient. Therefore, large and prospective trials are required to reveal the value of surgery for mPCa. There were some differences in case characteristics between the SEER and Chinese cohorts, which might be related to prevalence, economic status, religious beliefs, and eligibility for health insurance.

Many models for prognostic stratification based on epidemiological and clinicopathological features performed good clinical utility (23, 32). In our study, the nomogram was constructed based on the very large sample numbers from the SEER database, which suggested that results were reliable and stable. Through curve analysis, irrespective of the internal or external validation, the nomogram performed well in terms of discrimination and accuracy. In our study, the AUC values of the nomogram were all more than 0.7 in the training and validation cohorts. However, a large AUC and good agreement between predicted and observed results does not directly represent the clinical utility of the nomogram (33, 34). Thus, DCA, an advanced tool used to examine efficiencies of diagnostic tests and predictive models (35), was also performed in this study. The results demonstrated that the nomogram could perform well in terms of predictive efficiency and clinical application. Besides, histological sampling of the pancreatic primary tumor or liver metastases could provide a specific genetic signature to predict survival. Recent studies revealed that endoscopic ultrasound-guided fine-needle biopsy (EUS-FNB) had excellent diagnosis value in both solid pancreatic lesions and cystic pancreatic lesions (36–38). Even for mPCa, endoscopic ultrasound-guided fine-needle tissue acquisition (EUS-TA) could provide diagnosis and evaluation of liver metastases, which is the most common metastatic location of pancreatic cancer (39). EUS-TA not only played an important role in the diagnosis but also provided a new method for risk assessment and prognostic stratification of PCa (40). To be exact, EUS-TA was a cost-effective and efficient way to extract cells or tissues, especially for mPCa, which were used to perform predictive molecular marker and gene expression analyses. By combining the results of EUS-TA with epidemiological and clinicopathological features to create a more efficient model for risk assessment, it would benefit individualized treatment of mPCa.

Admittedly, there were several limitations to this study that cannot be ignored. First, some potential risk factors related to early death such as peritoneal metastases, unhealthy lifestyle habits (such as alcohol consumption and smoking), and history of past illness were lacking in the SEER database. Second, results might be influenced by selection bias from excluded and incomplete data. Third, although validation of the nomogram with an external cohort may help avoid overfitting of the model, the number of cases in the external validation cohort may have been insufficient. However, data from our center are well represented and fit the nomogram as external validation. Moreover, both internal and external validation cohorts confirmed the excellent applicability of this nomogram, indicating that the nomogram model is suitable for PCa patients of different races. For better external validation, large samples from international multicenter cohorts are desired.

In conclusion, a comprehensive and accurate nomogram based on risk factors associated with early mortality in mPCa was developed. The simple-to-use nomogram might provide oncologists with a suitable tool to devise individualized and precise treatment strategies, and could be further applied to improve survival outcomes for patients with mPCa.

## Data Availability Statement

Publicly available datasets were analyzed in this study. This data can be found here: https://seer.cancer.gov/data/.

## Author Contributions

ZZ and HZ contributed to the study design and literature search. ZZ and JP completed the data analysis. ZZ and JP generated and improved the figures and tables. ZZ completed the manuscript. JP and HZ proofread the manuscript. All authors contributed to the article and approved the submitted version.

## Funding

This research was supported by a grant from Jiangsu Provincial Medical Youth Talent (QNRC2016809).

## Conflict of Interest

The authors declare that the research was conducted in the absence of any commercial or financial relationships that could be construed as a potential conflict of interest.

## Publisher’s Note

All claims expressed in this article are solely those of the authors and do not necessarily represent those of their affiliated organizations, or those of the publisher, the editors and the reviewers. Any product that may be evaluated in this article, or claim that may be made by its manufacturer, is not guaranteed or endorsed by the publisher.

## Abbreviations

PCa, pancreatic adenocarcinoma; mPCa, metastatic pancreatic adenocarcinoma; DCA, decision curve analysis; CI, confidence interval; SEER, Surveillance, Epidemiology, and End Results; ICD-O-3, International Classification of Tumor Diseases Third Edition; AJCC, American Joint Commission on Cancer; ROC, receiver operating characteristic; AUC, area under the curve.
